# Ostomy-related problems and their impact on quality of life of colorectal cancer ostomates: a systematic review

**DOI:** 10.1007/s11136-015-1050-3

**Published:** 2015-06-30

**Authors:** Sylvia M. Vonk-Klaassen, Hilde M. de Vocht, Marjolein E. M. den Ouden, Eric Hans Eddes, Marieke J. Schuurmans

**Affiliations:** Research Centre for Elderly Care and Palliative Care, University of Applied Sciences Saxion, PO Box 70.000, 7500 KB Deventer, Enschede, The Netherlands; Deventer Ziekenhuis, Deventer, The Netherlands; Department of Rehabilitation, Nursing Science and Sports, University Medical Center Utrecht, Utrecht, The Netherlands; Faculty Chair Care for the Chronically Ill and Elderly, University of Applied Sciences Utrecht, Utrecht, The Netherlands

**Keywords:** Colostomates, Ostomy-related problems, Quality of life

## Abstract

**Aim:**

Many long-term ostomates are ‘out-of-sight’ of healthcare, and it is unknown how ostomates deal with ostomy-related problems and how these problems affect their quality of life (QOL). The aim is to examine patient-related studies describing ostomy-related problems and their impact on the perceived QOL of long-term colostomates.

**Methods:**

The electronic databases PubMed (MEDLINE), CINAHL, Cochrane Library and PsycINFO were systematically searched. All studies were included in which ostomy-specific QOL was measured using validated multidimensional instruments.

**Results:**

Of the 6447 citations identified, 14 prevailingly descriptive cross-sectional studies were included. Three different validated multidimensional instruments for measuring QOL in ostomates were used (EORTC C30/CR38, MCOHQOLQO, Stoma QOL Questionnaire). All studies demonstrated that living with a colostomy influences the overall QOL negatively. The ostomy-related problems described included sexual problems, depressive feelings, gas, constipation, dissatisfaction with appearance, change in clothing, travel difficulties, feeling tired and worry about noises.

**Conclusion:**

In conclusion, all 14 studies gave an indication of the impact of ostomy-related problems on the perceived QOL and demonstrated that a colostomy influences the QOL negatively. There is a wide range of ostomy-specific QOL scores, and there seem to be higher QOL scores in the studies where the MCOHQOLQO instrument was used. The MCOHQOLQO and the Stoma QOL Questionnaire gave the most detailed information about which ostomy-related problems were experienced. This review adds knowledge about the impact of stoma-related problems on QOL of long-term ostomates, but more research has to be conducted, to detect ostomy-related problems and especially possible care needs.

**Electronic supplementary material:**

The online version of this article (doi:10.1007/s11136-015-1050-3) contains supplementary material, which is available to authorized users.

## Introduction

Worldwide, colorectal cancer is the third most common type of cancer in men and the second in women with the highest incidence rates in North America, Australia, New Zealand, Europe and Japan. Colorectal cancer is primarily diagnosed in persons of 60 years and older [1]. Based on demographic trends, it is expected that the number of new patients with colorectal cancer will increase. Surgery, the most common treatment for colorectal cancer results in 10 % of the cases in a permanent ostomy. In 2011, 13.237 people in the Netherlands were diagnosed with colorectal cancer, and 1908 people got a permanent ostomy due to colorectal cancer (median age 71 years) [2, 3].

Several studies have shown that the overall complication rate after ostomy surgery is about 21–70 %, including late complications such as peristomal dermatitis, parastomal hernia, prolapse and stenosis [4]. Strikingly, some complications remain untreated for years, and a large group of ostomates is ‘out of sight’ of healthcare professionals. When complications arise, ostomates wait too long to contact healthcare professionals or do not contact them at all [5, 6]. It is unknown whether or how those ostomates deal with ostomy-related problems and how these problems might affect their quality of life (QOL).

Grant et al. [7] found that several studies reveal that the presence of an intestinal stoma is an important QOL concern for both cancer and non-cancer patients. At the same time, it is widely recognized that QOL is difficult to measure. Wilson and Cleary [8] state that QOL is the subjective evaluation of one’s personal satisfaction with overall health and well-being. It is an important outcome of cancer survivorship that includes QOL related to physical, functional, psychological and social functioning. QOL instruments focusing on the generic QOL are not sensitive enough to detect the specific impact an ostomy has on an ostomates’ QOL [7]. With a multidimensional QOL instrument, focusing on the effects of an intestinal stoma, specific areas of concern of ostomates can be identified. They include physical well-being and symptoms, psychological well-being, social well-being and spiritual well-being.

The current systematic review focuses on patient-related studies in which specific ostomy-related problems and their impact on the perceived QOL of long-term ostomates are described. The following research questions will be addressed:

1. What is the perceived ostomy-specific quality of life of long-term ostomates, with a colostomy due to colon rectal cancer?
2. Which ostomy-related problems affect the perceived ostomy-specific quality of life of these ostomates?

The discussion of findings will be presented in relation to what future studies are needed, both quantitative and qualitative, to further describe long-term ostomy-related problems and their impact on quality of life.

## Methods

### Search strategy

**Fig. 1:**
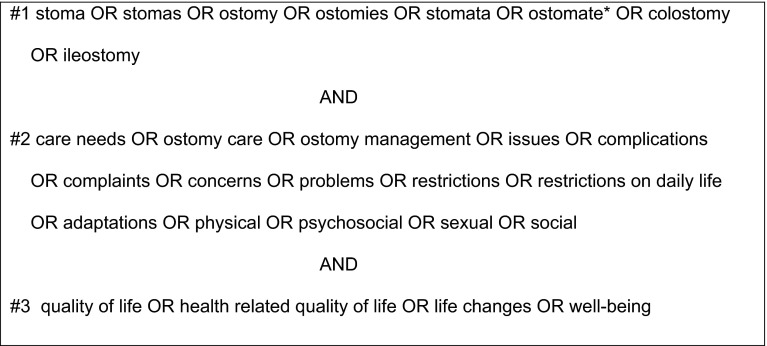
Search strategy

The electronic databases PubMed (MEDLINE), CINAHL, Cochrane Library and PsycINFO were systematically searched for studies assessing ostomy-related problems, care needs and quality of life experienced by long-term ostomates. Outcomes of interest were: quality of life and ostomy-related problems. Figure 1 shows the search terms that were used in combination.

Search terms related to ‘long-term’ were not included as this resulted in too many limitations in the number of relevant results (i.e., not all relevant publications specified the time since construction of the ostomy). The search was limited to the English language. There was no restriction on year of publication. The references of the included articles were scanned to find other relevant studies.

### Selection criteria

The following inclusion criteria were defined for the present study: articles reporting data of an original study, studies including a population with a permanent ostomy due to colorectal cancer, adults, long-term ostomates (beyond the first year after surgery) and measurement of ostomy-specific QOL with a validated multidimensional instrument. Qualitative studies, unpublished studies, abstracts, dissertations, theses and book chapters are excluded for the present review.

### Selection process

**Fig. 2:**
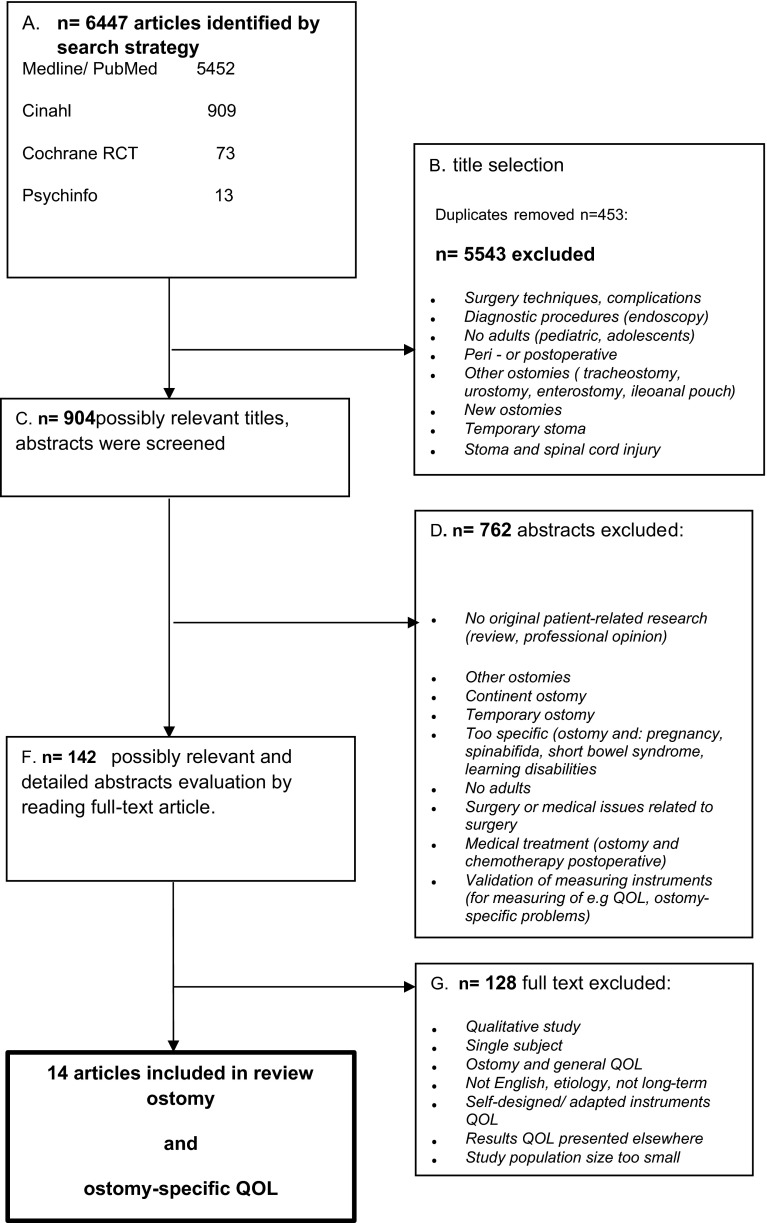
Flow diagram of the study selection process

Figure 2 shows the flow diagram of the study selection process. Firstly, all titles (*n* = 6447) were checked based on the criteria ostomy-related problems and quality of life in combination with long-term ostomates independently by two researchers (SV and MDO). Secondly, abstracts of the 904 possibly relevant titles were screened using the inclusion criteria, resulting in 142 possibly relevant studies. Thirdly, of these 142 abstracts, the full-text articles were read and another 128 were excluded. In total, 14 studies met the inclusion criteria.

### Methodological quality

The assessment of the methodological quality of the studies included in this review was based on an adapted 13-item version of a 14-item checklist for systematic reviews, developed by Mols et al. [9] for a systematic review about QOL among long-term breast cancer survivors. This instrument is also used in other systematic reviews concerning QOL [10, 11]. See supplement I for the checklist. For each study, one point for each item is assigned if it matches the criteria of the checklist, and zero points if it does not. The maximum score is 13 points. The studies scoring 75 % or more of the maximum score, i.e., 10–13 points, are considered to be of high quality. Studies scoring between 50 and 75 % are rated as moderate quality. Studies scoring lower than 50 % are rated as low quality and will be excluded for this review.

### Data analysis

To make a comparison of the data from the three different QOL instruments possible and present them in one overview, all QOL scores were linearly transformed to a 0–100 scale. A high score represents a higher level of QOL and a lower level of symptoms.

## Results

### Search results

In total, 14 studies were included. The majority of the studies were descriptive cross-sectional studies (*n* = 12), and the remaining studies had a longitudinal design (*n* = 2). Three validated multidimensional instruments for measuring quality of life in ostomates were used (see supplement II for a brief description of the instruments):

- EORTC C30/CR38, European Organisation for Research and Treatment of Cancer (*n* = 10) [12].
- MCOHQOLQO, Modified City of Hope Colorectal Cancer Quality of Life Questionnaire Ostomy (*n* = 3) [7].
- Stoma Quality of Life Questionnaire (*n* = 1) [13].

The EORTC C30 in combination with the EORTC CR38 is developed for colorectal cancer patients including ostomates. QOL is one specific question in C30 and relates to QOL for colorectal cancer patients in general. A high score on a symptom scale in CR38, such as stoma-related problems, represents a high level of symptomatology and problems and consequently worse QOL. The MCOHQOLQO and Stoma Quality of Life Questionnaire are developed for ostomates. In these questionnaires, QOL is calculated as the sum of the scores on several ostomy-related items. The baseline characteristics of the 14 studies included in this review are shown in Table 1. The overall methodological quality of the included studies ranged from 11 to 13 points (supplement III), so no studies were excluded based on low methodological quality.

**Table 1:** Baseline characteristics of the included studies

Authors	Design	crc *n*	Participants					QOL instrument	Subject study	Meth score
			Colost *n*	Recruited	Time since surgery	Age years (SD) or [range]	% women			
Bloemen et al. [14]	C	121	51	2 hos	36 months	67 [32–96]	42	EORTC C30CR38	QOL and postoperative complications and ostomy	11
Anaraki et al. [25]	C	102	69	Ost As	>1 year	53.5 (12.3)	43.1	MCOHQOLQO	QOL and living with an ostomy	11
Camilleri-Brennan and Steele [15]	C	106	71	4 hos	AR 26 months AP 29 months	AR 68 AP 71	49	EORTC C30CR38	QOL, morbidity and surgery techniques AR/AP	12
Krouse et al. [26]	C	491	246	3 regions	12 years	72.4 (10.3)^a^	40.2^a^	MCOHQOLQO	QOL and manifestations by sex	12
Orsini et al. [16]	C	143	67	4 hos/ca region	3.4 years	64.7 (11.1)^a^	38.7	EORTC 38/SF36	QOL and stoma/older people	12
Krouse et al. [24]	C	599 colo	517 ca	Ost As	135.9 months	72.5 (10.4) (ca)^a^	51 (ca)	MCOHQOLQO	QOL and ostomy and cancer/no cancer	11
Kald et al. [27]	C	70	70	1 hos	8.1 years	71.7 (13.7)	54	Stoma QOL Q	QOL and stoma with/without bulging	11
Hoerske et al. [17]	C	219	22	1 hos	13 years	70 [41–94]	40	EORTC C30CR38	QOL and RC surgery/need for a ostomy	12
Konanz et al. [18]	C	131	50	1 hos	59 months	69.2 (11.5)^a^	24	EORTC C30CR38	QOL and RC surgery methods	12
Fucini et al. [19]	C	62	30	1 hos	5 years	70 [13]^a^	36	EORTC C30CR38	QOL and treatment and with/without ostomy	11
Arndt et al. [20]	L	222	38	1 region	>2 years	66 (9.2)	48	EORTC C30CR38	Restrictions in QOL and colorectal cancer	12
Mahjoubi et al. [21]	C	348	348	Ost As	<2 years	53.2* (nm)^a^	58*^a^	EORTC C30CR38	QOL and stoma site and problems	11
Mahjoubi et al. [22]	C	96	96	1 hos	32 months	48.8 (1.49)	54	EORTC C30CR38	QOL and colostomy and age, gender difference	11
Engel et al. [23]	L	299	77	ca region	1–4 years	212 < 70 87 > 70 (nm)	64.2	EORTC C30CR38	QOL and RC patient and surgery	12

Nm = not mentioned; design: L = longitudinal, C = cross-sectional; age: age of whole population or ^a^ = of ostomates, (SD) [range]; % women: of whole population or ^a^ = of ostomates

ca = cancer/crc = colorectal cancer/colo = colostomy/ileo = ileostomy/rc = rectal cancer/ca region = cancer registration/hos = hospital/ost as = ostomy association/*appropriate stoma site/AR = anterior resection/AP = abdominoperineal resection

### Population

In most studies (*n* = 10), the colostomates (range *n*: 22–517) were part of a population of colorectal cancer patients [14–23]. The average age is about 61 and varies between 48.8 and 72.5 years. The post-treatment period ranges from 1 to 12 years. The participants of all studies participated voluntarily and were recruited from a hospital, the Ostomy Association or Cancer Registry (Table 1).

### Outcomes of the studies

#### Ostomy-specific QOL

In this overview of ostomy-specific QOL (Table 2), all QOL scores are based on self-reported ostomy-related problems. A high score represents a higher level of QOL and a lower level of symptoms.

**Table 2:**
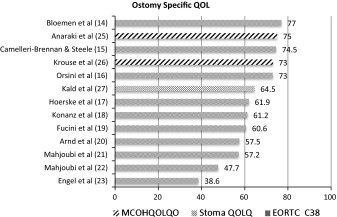
QOL based on self-reported ostomy-related problems

In another study of Krouse et al. [24] the Ostomy-Specific QOL 32 % of the population (*N* = 517) scored <7, on the ostomy-specific QOL, indicating moderate-to-severe QOL (MCOHQOLQO). These outcomes (described in percentage) could not be included in Table 2 as the information required was not presented in the publication.

#### Relation ostomy-related problems and QOL

In 10 studies [14–23], the EORTC C38 was used to measure QOL. The ostomy-related problems are scored as one item, and the studies did not describe the outcome per sub-item (i.e., *afraid about stoma, noise, afraid about smell of stools, worry about possible leakage, caring for stoma, irritated skin, embarrassment, feeling less complete*). Hence the EORTC C38 score (range 38.6–77) is an indication of the overall impact of ostomy-related problems on QOL.

In 3 studies [24–26], the MCOHQOLQO was used to measure QOL. A total QOL measure (range 73–75) was computed by summing scores on all items of the four dimensions (*physical well*-*being, psychological well*-*being, social well*-*being and spiritual well*-*being*). The ostomy-related problems mentioned included sexual problems, feeling depressed, gas, constipation, dissatisfaction with appearance, change in clothing, travel difficulties.

Anaraki et al. [25] concluded that living with a stoma influences overall QOL. About 70 % of the patients were dissatisfied with ‘sexual activity’ and ‘depression feelings.’ Factors such as the type of ostomy (temporary/permanent), the underlying disease that had led to the stoma, depression, problem with location of ostomy, change in clothing had significant effects on overall QOL and its subscales (*p* < 0.05). In the study of Krouse et al. [24], QOL of cancer and non-cancer patients with ostomies was studied and their results confirmed the negative impact of a colostomy on QOL. Of the patients with cancer, 32 % reported moderate-to-severe-QOL concerns (<7 on scale of 0–10, where 10 is the highest QOL). On the subscales, patients also scored <7, namely: physical 22 %, psychological 37 %, social 25 % and spiritual 32 %. While patients with cancer had a better overall QOL compared to patients with benign processes (<7: cancer 32 % vs. non-cancer 48 %) and less difficulty adjusting to their ostomies, concerns were common to all colostomy patients for example sexual problems, gas, constipation, travel difficulties and dissatisfaction with appearance. In another study of Krouse et al. [26], the subject of the study was health-related QOL among long-term colorectal cancer survivors with an ostomy and manifestations by gender. Both men and women had significantly worse social well-being compared to controls (men: mean-adjusted difference (MAD): −0.88, *p* < 0.01; women: MAD = −1.16, *p* < 0.01), with only female cases reporting significantly worse overall HRQOL (MAD: −0.72; *p* < 0.02) and psychological well-being (MAD: −0.93, *p* < 0.01). Men and women report a different profile of challenges, suggesting the need for targeted or gender-specific interventions to improve HRQOL in this population such as a focus on physical HRQOL for female ostomy survivors younger than age 75.

In one study, the Stoma Quality of Life Questionnaire was used to measure QOL (score 64.5). QOL was based on four domains: sleep, sexual activity, relations to family and close friends and social relations with others than family and close friends (20 items). In this study of Kald et al. [27], the QOL in ostomates with and without bulging was measured. Most items scored (median answers in patients with normal finding and with bulging) ‘Rarely’ or ‘Not at all’; none scored ‘Always.’ The items which scored ‘Sometimes’ are: *I need to know where the nearest toilet is (bulging), I feel tired during the day (bulging), I need to rest during the day (bulging), I worry about noises from the stoma (bulging and normal finding).* There was a small but statistically significant difference between patients with and without bulging, but they stated that further studies are required to evaluate the role of some of the individual items in the Stoma Quality of Life Questionnaire.

### Summary of findings

In conclusion, all 14 studies gave an indication of the impact of ostomy-related problems on the perceived QOL, and all studies demonstrated that living with a colostomy influences the overall QOL negatively. There is a wide range of scores for ostomy-specific QOL (38.6–77), and there seem to be higher QOL scores in the studies using the MCOHQOLQO instrument (range 73–75). The MCOHQOLQO and the Stoma Quality of Life Questionnaire gave the most detailed information about which ostomy-related problems were experienced. The ostomy-related problems mentioned included sexual problems, feeling depressed, gas, constipation, dissatisfaction with appearance, change in clothing, travel difficulties, feeling tired, worry about noises from the ostomy.

Many factors might have an effect on ostomy-specific QOL, such as age, gender, time since treatment, but in this review there was no conclusive evidence for any of these factors.

When comparing the longitudinal studies, on the one hand, Arndt et al. [20] found scores of 54.2 and 57.5 (at 1 and 3 years post-treatment), which implies less problems and higher QOL over time. On the other hand, Engel et al. [23] found scores of 46.7 and 38.6 (at 1 and 4 years post-treatment) which implies more problems and lower QOL in time. No explanation about the contradicting findings can be given from the baseline characteristics of the studies.

## Discussion and conclusion

The aim of the present review was to examine patient-related studies describing ostomy-related problems and their impact on the perceived QOL of long-term colostomates. Fourteen prevailingly descriptive, cross-sectional studies were included. Three different validated multidimensional instruments for measuring QOL in ostomates were used. All studies demonstrated that living with a colostomy influences the overall QOL negatively. The ostomy-related problems mentioned included sexual problems, depression feelings, gas, constipation, dissatisfaction with appearance, change in clothing and travel difficulties, feeling tired and worry about noises from the ostomy. The instruments that were designed especially for ostomates gave the most detailed information.

All 14 studies were of good methodological quality (10–13 points) and gave an indication of the impact of stoma-related problems on the perceived QOL. Not all studies described the sub scores of the ostomy-related problems, which would have led to more detailed information instead of just one number. Furthermore, each QOL instrument has different parameters, making a direct comparison impossible. In all studies, the population (range *n* 22–517) was part of a colorectal cancer population and volunteered to participate in the study. It is not clear whether these study population are representative for the entire population of long-term ostomates and especially the ‘out-of-sight’ population.

### Reviews

A few reviews on the subject QOL and having colorectal cancer (with or without an ostomy) are available; however, they mostly focus on the general QOL of all CRC patients including those who are having an ostomy [11, 28–30]. In the study of Pachler and Wille-Jorgeson [30], ostomates were compared with non-ostomates; in the other two reviews, the ostomates were part of a colorectal cancer population. In all these reviews, the used instruments to measure QOL in the studies were generic, disease specific and incidental ostomy specific (MCOHQOLQO).

In the Cochrane review of Pachler and Wille-Jorgeson [30], the general QOL of rectal cancer patients with or without a permanent colostomy was compared. In their review, they included 26 articles, all clinical controlled trials and observational studies in which quality of life was measured in patients with rectal cancer with or without the construction of a stoma, using a validated QOL instrument. They concluded that there were no apparent differences in QOL of life found between the two groups. However, they also mentioned that it was not possible to draw definite conclusions because of the many different instruments used, the different types of study (retrospective or prospective) and different time periods (early or late postoperative).

Denlinger et al. [28] found that the presence of a permanent ostomy may affect QOL and a permanent ostomy has been associated, for example, with diminished body image and increased financial worries although global quality of life was not affected. Jansen and Koch [11] describe in their review that survivors with ostomies had more problems in physical and role functioning, worse scores on fatigue, dyspnea and appetite loss, and more body image and sexual functioning problems than non-ostomy survivors.

### Other research

To get more detailed information about the perceived ostomy-related problems, qualitative research is more suitable. A few qualitative studies were carried out which provide more detailed ‘in-depth’ information about the perceived problems and possible care needs on all domains [31–34]. The population size in all studies varied from 14 to 178, and patients (mostly long-term ostomates) volunteered to participate. In 3 of the 4 studies, the same cohort was used.

In the study of Grant et al. [31], about gender differences in QOL between long-term colorectal cancer survivors with ostomies common issues included diet management, physical activity, social support and sexuality. Women described more specific psychological and social issues than men. In the study of Sun et al. [32], about long-term persistent ostomy-specific concerns and adaptations, they concluded that persistent ostomy-related issues more than 5 years after formation were common. Persistent ostomy-related issues were focused on clothing restrictions and adaptations, dietary concerns, issues related to ostomy equipment and self-care, and the constant need to find solutions to adjust and readjust to living with an ostomy. Adaptations tend to be individualized and based on trial and error. These findings underscore the need to develop long-term support mechanisms in that survivors can access to promote better coping and adjustment to living with an ostomy. The study of McMullen et al. [33] reported the greatest challenges reported by long-term colorectal cancer survivors with ostomies. For survivors of colorectal cancer who have a permanent colostomy or ileostomy, permanent physical changes in bowel functioning require daily care adjustments and challenging psychological and social adaptations. Dabirian et al. [34] explored quality of life in ostomy patients. In this study, nine main themes emerged: physical problems related to colostomy, impact of colostomy on psychological functioning, social and family relationships, travel, nutrition, physical activity, sexual function, religious and economic issues.

Furthermore, a study was conducted by Lynch et al. [35] about ostomy surgery for colorectal cancer: a population-based study of patient concerns (*n* = 332 of which 109 permanent colostomies). They concluded that a painful or irritated peristomal skin and odor and noise from the appliance were the most commonly reported ostomy-related difficulties. The proportion of participants reporting these difficulties decreased over time. Provision of preoperative information was comprehensive, and satisfaction with preoperative information was high. However, 34 % of patients said they were not seen by an ostomy nurse prior to surgery.

One of the questions that remains is why complications remain untreated for years. And how (if at all), did the ostomate recognize the problem, how long did the problem persist and what was the necessary or desired care need. Inadequate treatment of ostomy-related problems, such as skin complications, not only can have an adverse impact on quality of life but will also increase treatment costs [5].

## Conclusion

This review adds knowledge about the impact of ostomy-related problems on QOL of long-term ostomates. In all articles, included in the review and other research mentioned in the discussion, ostomy-related problems were described on all domains of ostomy-specific QOL. The qualitative research provided more detailed information about the problems, unmet needs and ways ostomates adapted [31], demonstrating that adaptations tend to be individualized and based on trial and error [32]. In addition, an ostomy can change over the years, and therefore, the experienced problems can vary over time.

An intriguing point is the fact that although the ostomy-specific QOL was often acceptable, there were still a number of ostomy-related problems that needed to be solved. The studies using the MCOHQOLQO, the most comprehensive instrument, revealed higher QOL scores despite many ostomy-related problems. More research, qualitative and quantitative, has to be conducted, to detect more information about the ostomy-related problems and especially possible care needs (prevention, detection, treatment).

## Electronic supplementary material

Supplementary material 1 (DOCX 15 kb)

Supplementary material 2 (DOCX 15 kb)

Supplementary material 3 (DOCX 17 kb)
