# Supplementary material for: Ostomy-related problems and their impact on quality of life of colorectal cancer ostomates: a systematic review
Source: Qual Life Res. 2015 Jun 30;25:125–33. doi: 10.1007/s11136-015-1050-3 (PMC4706578; doi:10.1007/s11136-015-1050-3)
Supplement: Supplementary file 1 — Supplementary material 1 (DOCX 15 kb) [file 11136_2015_1050_MOESM1_ESM.docx]

**Supplement I** Criteria list for assessing the methodological quality of studies

| **The assessment of the methodological quality was based on a 14-item checklist for systematic reviews, developed by Mols et al (9) and is adapted for this review. Each item of a selected study, is assigned 1 point if it matches the criteria of the checklist, and 0 points if it doesn’t. The highest possible score is 13 points. The studies scoring 75% or more of the maximum score, 10-13 points, are considered to be of high quality. Studies scoring between 50% and 75%, are rated as moderate quality, and studies scoring lower than 50% as low quality.** | |
| --- | --- |
|  | Socio-demographic and medical data is described (e.g., age, gender, diagnosis etc..) |
|  | Inclusion and/or exclusion criteria are formulated |
|  | The process of data collection is described (e.g., survey interview or self-report etc.) |
|  | The type of cancer treatment is described |
|  | The results are compared between two groups or more (e.g., healthy population, groups with or without ostomy or age, comparison with time at diagnosis etc.) |
|  | Time since diagnosis or treatment is given |
|  | Participation and response rates for patient groups have to be described and have to be more than 75% |
|  | Information is presented about patient/disease characteristics of responders and non-responders or if there is no selective response |
|  | A standardized or valid quality of life questionnaire is used (measuring ostomy- quality of life) |
|  | Results are not only described for quality of life but also for the physical, psychological and social domain |
|  | Mean, median, standard deviations or percentages are reported for the most important outcome measures |
|  | Patient signed an informed consent form before study participation |
|  | The degree of selection of the patient sample is described |
