# Supplementary material for: Ostomy-related problems and their impact on quality of life of colorectal cancer ostomates: a systematic review
Source: Qual Life Res. 2015 Jun 30;25:125–33. doi: 10.1007/s11136-015-1050-3 (PMC4706578; doi:10.1007/s11136-015-1050-3)
Supplement: Supplementary file 2 — Supplementary material 2 (DOCX 15 kb) [file 11136_2015_1050_MOESM2_ESM.docx]

**Supplement II** Description of the QOL instruments

| **EORTC** |
| --- |
| **C30** : Assesses QOL in cancer patients, including 30 items of which five functional scales (physical PF, role RF, emotional EF, cognitive CF and social functioning SF), three symptom scales (fatigue FA, pain PA and nausea and vomiting NV) and one global health status and quality of life scale. IN addition, six single items ( dyspnea DY, insomnia IN, appetite loss AP, constipation CO, diarrhea DI, and financial difficulties FI). **CR 38:** Developed specifically for rectal cancer patients including 38 questions, which quantifies QOL using four functional scales ( body image BI, future perspective FU, sexual activity SX, and sexual enjoyment SE) and eight symptom scales (micturion MI, gastrointestinal disorders GIT, systematic therapy side effects CT, male sexual problems MSX, Female sexual problems FSX, weight loss WL, stoma-related problems STO. *(Stoma-related problems are scored as one item, the several sub items are not shown in the articles: Afraid about stoma, Noise, Afraid about smell of stools, Worry about possible leakage, Caring for stoma, Irritated skin, Embarrassment, Feeling less complete)* **C30 and CR38**: The scores range from 0-100. A higher score on a functional scale represents a high level of functioning and higher QOL, whilst a high score on a symptom scale or item represents a high level of symptomatology and problems and worse QOL |
| **modified City of Hope Quality of Life Questionnaire Ostomy (mCOHQOLQO)** |
| Assesses QOL in patients with an ostomy (Grant, 2003) The questionnaire is composed of two sections, The first section contains 46 descriptive questions used to identify demographics of the disease, treatment, ostomy specific and other personal characteristics including diet, work, and activity. The second section is composed of 43 items, divided into the four dimensions: physical well- being, psychological well-being, social well-being, spiritual well-being. Items under each dimension are scaled from 0-10, with 0 being the worst QOL and 10 being the best QOL. Overall scores for each dimension are computed by summing items in each subscale and dividing by the number of items in that subscale. Likewise, a total QOL measure is computed by summing scores on all items and dividing by the total number of items |
| **Stoma Quality of Life Questionnaire** |
| Stoma Quality of Life Questionnaire (Prieto, 2005) is a cross-cultural QoL instrument which is developed and validated specifically for patients with a colostomy of ileostomy and is based on the needs-based model of QOL of Hunt and McKenna’s. There are four domains: sleep, sexual activity, relations to family and close friends and social relations with others than family and close friends. The 20 items in the Stoma QOLQ can be answered on a 4-point scale: 1-Always, 2- Sometimes, 3- Rarely, 4- Not at all. Scores for each of the 20 items have different weights resulting in a final score of 0-100, where 100 indicates the best possible QoL and 0 the worst QoL . The Stoma QoL Q has been translated into and validated in English, Danish, Dutch, German, French and Spanish. |
