# Supplementary material for: Ostomy-related problems and their impact on quality of life of colorectal cancer ostomates: a systematic review
Source: Qual Life Res. 2015 Jun 30;25:125–33. doi: 10.1007/s11136-015-1050-3 (PMC4706578; doi:10.1007/s11136-015-1050-3)
Supplement: Supplementary file 3 — Supplementary material 3 (DOCX 17 kb) [file 11136_2015_1050_MOESM3_ESM.docx]

**Supplement III** Assessing Methodological Quality of the included studies (adapted from Mols et al (9)

| ***Study*** | ***A*** | ***B*** | ***C*** | ***D*** | ***E*** | ***F*** | ***G*** | ***H*** | ***I*** | ***J*** | ***K*** | ***L*** | ***M*** | ***Method Score*** |
| --- | --- | --- | --- | --- | --- | --- | --- | --- | --- | --- | --- | --- | --- | --- |
| Bloemen et al (14) | *1* | *1* | *1* | *11* | *1* | *1* | *0* | *1* | *1* | *1* | *1* | *0* | *1* | ***11*** |
| Anaraki et al (25) | *1* | *1* | *1* | *1* | *1* | *0* | *1* | *0* | *1* | *1* | *1* | *1* | *1* | ***11*** |
| Camilleri-Brennan & Steele (15) | *1* | *1* | *1* | *1* | *1* | *1* | *1* | *0* | *1* | *1* | *1* | *1* | *1* | ***12*** |
| Krouse et al (26) | *1* | *1* | *1* | *1* | *1* | *1* | *0* | *1* | *1* | *1* | *1* | *1* | *1* | ***12*** |
| Orsini et al (16) | *1* | *1* | *1* | *1* | *1* | *1* | *1* | *0* | *1* | *1* | *1* | *1* | *1* | ***12*** |
| Krouse et al (24) | *1* | *1* | *1* | *1* | *1* | *1* | *0* | *0* | *1* | *1* | *1* | *0* | *1* | ***11*** |
| Kald et al (27) | *1* | *1* | *1* | *1* | *1* | *1* | *1* | *0* | *1* | *1* | *1* | *0* | *1* | ***11*** |
| Hoerske et al (17) | *1* | *1* | *1* | *1* | *1* | *1* | *1* | *1* | *1* | *1* | *1* | *0* | *1* | ***12*** |
| Konanz et al (18) | *1* | *1* | *1* | *1* | *1* | *1* | *1* | *1* | *1* | *1* | *1* | *0* | *1* | ***12*** |
| Fucini et al (19) | *1* | *1* | *1* | *1* | *1* | *1* | *1* | *0* | *1* | *1* | *1* | *1* | *1* | ***11*** |
| Arndt et al (20) | *1* | *1* | *1* | *1* | *1* | *1* | *0* | *1* | *1* | *1* | *1* | *1* | *1* | ***12*** |
| Mahjoubi et al (21) | *1* | *1* | *1* | *1* | *1* | *1* | *1* | *0* | *1* | *1* | *1* | *0* | *1* | ***11*** |
| Mahjoubi et al (22) | *1* | *1* | *1* | *1* | *1* | *1* | *1* | *0* | *1* | *1* | *1* | *0* | *1* | ***11*** |
| Engel et al (23) | *1* | *1* | *1* | *1* | *1* | *1* | *0* | *1* | *1* | *1* | *1* | *1* | *1* | ***12*** |
